# Emerging Trends in Chitin-Based Hydrogels: From Fundamental Properties to Advanced Applications

**DOI:** 10.3390/gels12040321

**Published:** 2026-04-09

**Authors:** Merreta Noorenza Biutty, Ratri Puspita Wardani, Zeno Rizqi Ramadhan, Boram Yun, Achmad Yanuar Maulana, Jongsik Kim, Maulida Zakia

**Affiliations:** 1 Department of Chemical Engineering, Faculty of Industrial Engineering, Universitas Pembangunan Nasional “Veteran” Yogyakarta, Yogyakarta 55283, Indonesia; 2 Department of Chemical Engineering, Faculty of Engineering and Science, Universitas Pembangunan Nasional “Veteran” Jawa Timur, Surabaya 60294, Indonesia; 3 School of Chemistry, The University of New South Wales, Sydney, NSW 2052, Australia; 4 Department of Chemical Engineering (BK21 FOUR Graduate Program), Dong-A University, Busan 49315, Republic of Korea; 5 Department of Chemistry, Dong-A University, Busan 49315, Republic of Korea; 6 DAU G-LAMP Project Group, Institute of Natural Science, Dong-A University, Busan 49315, Republic of Korea; 7 DAU G-LAMP Project Group, Innovation of Center for Atomic Science, Dong-A University, Busan 49315, Republic of Korea; 8 Department of Chemical Engineering, Faculty of Engineering, Universitas Negeri Semarang, Semarang 50229, Indonesia

**Keywords:** chitin, hydrogels, stimuli-responsive materials, sustainable materials, biodegradable materials

## Abstract

Chitin-based hydrogels have emerged as a versatile and sustainable material with significant potential in biomedical, environmental, and energy applications. Derived from the abundant biopolymer chitin, these hydrogels exhibit exceptional biocompatibility, biodegradability, and tunable physicochemical properties. This review highlights advances in chitin-based hydrogels, focusing on solvent systems, crosslinking strategies, and structural modifications to enhance mechanical strength, swelling, and stimuli responsiveness. Key applications include wound healing, drug delivery, tissue engineering, and environmental remediation, where their high-water retention, enzymatic degradability, and eco-friendly nature are particularly advantageous. Furthermore, innovations such as nanoparticle incorporation and chemical derivatization (e.g., carboxymethylation, hydroxypropylation) have expanded their utility in energy devices and smart sensors. Despite these advances, challenges remain in optimizing the energy efficiency of production methods for industrial scalability. This review provides a comprehensive overview of the current state of chitin-based hydrogels, offering insights into future directions for research and development in this promising field.

## 1. Introduction

Hydrogels are three-dimensional polymer networks characterized by their remarkable capacity to absorb and retain a significant fraction of water while remaining insoluble in aqueous environments [1]. Their high-water uptake capacity is attributed to the presence of hydrophilic functional groups along the polymer backbone. These moieties interact with water molecules through hydrogen bonding and osmotic pressure, facilitating extensive water uptake [2]. In addition, their insolubility is maintained due to covalent bonding from chemical crosslinking or physical forces such as hydrogen bonding, ionic interactions, and crystalline phase formation, which stabilize the three-dimensional matrix and prevent dissolution.

The classification of hydrogels is generally based on the source of the polymeric materials used in their synthesis [3]. Hydrogels may be derived from natural biopolymers or synthetic polymers, each offering distinct advantages depending on the intended application. Natural biopolymer-based hydrogels are generally favored for their excellent biocompatibility, biodegradability, and inherent bioactivity, making them particularly suitable for biomedical applications such as wound healing [4,5,6,7], tissue engineering [8,9,10,11,12], and drug delivery [13,14,15]. These materials often exhibit minimal cytotoxicity and can mimic components of the extracellular matrix, enhancing cell attachment and proliferation. However, their mechanical properties and structural uniformity may be limited due to variability in natural sources and sensitivity to processing conditions [16]. In comparison, synthetic polymer-based hydrogels provide superior mechanical strength, structural stability, and reproducibility. They can be precisely engineered to achieve specific physical and chemical properties, such as tunable porosity, swelling behavior, and degradation rates. This level of control makes synthetic hydrogels highly suitable for applications requiring consistent performance and mechanical durability, including soft robotics, sensors, and controlled-release systems. However, they are generally characterized by low biodegradability and limited biocompatibility. Therefore, the selection between natural and synthetic hydrogel materials is often dictated by the specific performance requirements of the target application, balancing the need for biocompatibility with the demand for mechanical integrity and processability.

Among a wide range of natural biopolymers, chitin has emerged as a promising candidate for hydrogel development due to its unique structural and functional properties [17]. Structurally, chitin is a linear polysaccharide composed of β-(1→4)-linked N-acetyl-D-glucosamine units, and it is widely found in nature, particularly in the exoskeletons of crustaceans, insect cuticles, and fungal cell walls. Functionally, chitin-based hydrogels exhibit favorable properties such as high-water retention, biocompatibility, biodegradability, and hemostatic activity. These characteristics make them particularly suitable for biomedical applications. Their capacity to support cell adhesion and modulate drug release profiles further enhances their performance in therapeutic contexts. From a sustainability perspective, chitin offers the added advantage of being derived from renewable and underutilized seafood byproducts, making it an eco-friendly and cost-effective option for the development of green hydrogel materials. Therefore, this review presents an overview of the fundamental properties of chitin as a basis for hydrogel development, along with recent advances in preparation strategies in-volving native chitin, its derivatives, and nanochitin systems. It further discusses the key physicochemical characteristics governing their performance and highlights their multi-functional applications, including superabsorbents, controlled delivery systems, stimuli-responsive systems, energy devices, smart sensors, and tissue engineering, followed by concluding remarks and future perspectives.

## 2. Chitin

Chitin is one of the most abundant natural polysaccharides and the second most prevalent biopolymer on Earth, following cellulose. It is a naturally occurring mucopolysaccharide that serves as the primary structural component in the exoskeletons of crustaceans, insects, and other arthropods. Although chitin is widely distributed in nature, its primary commercial sources to date have been limited to the exoskeletal waste of marine crustaceans, particularly crab and shrimp shells. Despite its abundance, only a small fraction of extracted chitin is utilized in value-added applications, while the majority remains underexploited. Due to its inherent biocompatibility, biodegradability, non-toxicity, and ability to be chemically modified, chitin has attracted considerable attention as a renewable feedstock for the development of advanced biomaterials in biomedical [18,19], environmental [20], and energy-related fields [21].

**Figure 1:**
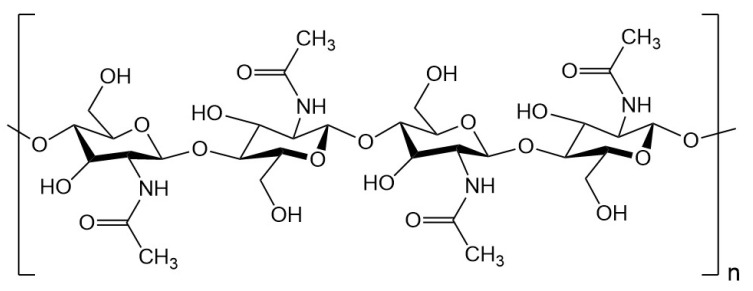
Chemical structure of chitin poly(N-acetyl-β-d-glucosamine).

Structurally, chitin is composed of repeating units of 2-acetamido-2-deoxy-β-D-glucopyranose, connected via β-(1→4) glycosidic linkage (Figure 1). The repeating monomer, 2-acetamido-2-deoxy-β-D-glucopyranose, forms the backbone of chitin, which shares structural similarity with cellulose but differs in the presence of acetamido groups that impart unique physicochemical characteristics. The degree of acetylation and molecular arrangement of chitin largely determine its crystallinity, solubility, and reactivity, which subsequently influence its biological function and potential for modification [22]. Chitin exists in three polymorphic forms—α, β, and γ—depending on the orientation of the polymer chains, with α-chitin being the most abundant and stable form found predominantly in crustacean shells. The polymer contains numerous functional groups, particularly hydroxyl and acetamido groups, which serve as reactive sites for chemical modifications such as deacetylation, carboxymethylation, or grafting, enabling the transformation of chitin into chitosan or other derivatives with tailored properties. Biotechnological advances have facilitated the enzymatic or microbial transformation of chitin into functional oligomers and nanostructures under mild and sustainable conditions [23,24,25]. These modified chitin-based materials exhibit enhanced bioactivity, mechanical properties, and processability, making them suitable for diverse applications, including drug delivery, tissue engineering, biosensing, and water treatment. Furthermore, amid escalating environmental and economic constraints linked to petroleum-derived polymers, chitin has emerged as a renewable, biodegradable biopolymer with significant potential for the development of high-performance bio-functional materials. Consequently, chitin has garnered significant attention for its potential in replacing synthetic polymers across various sectors, offering eco-friendly pathways to produce advanced biomaterials, bioplastics, membranes, and biomedical devices.

## 3. Preparation of Chitin-Based Hydrogels

Due to the low solubility properties of natural chitin, chitin-based hydrogels can be prepared through several ways. It can be crosslinked from the native chitin dissolved in particular solvents, derivatives of chitin dissolved in water or acidic solution, and nano chitin dissolved in aqueous solution.

### 3.1. Preparation of Hydrogels from Native Chitin

**Figure 2:**
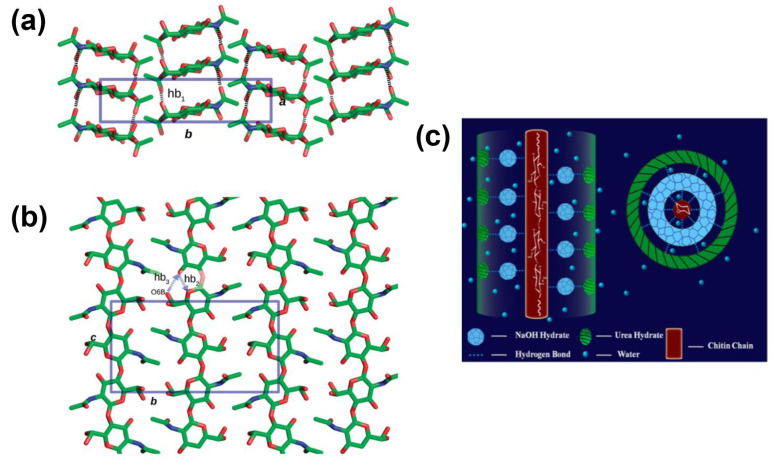
Crystalline configurations of α-chitin and β-chitin. (**a**) Top-to-bottom vertical views along the ab- and (**b**) bc- planes of the α-chitin structure at 100 K (Reproduced with permission from [26]). (**c**) A structural model of chitin complex chain in an aqueous NaOH/urea system (reproduced with permission from [27]).

The cross-linkage of chitin in solutions has limitations because of its high crystallinity properties. The crystallinity can be seen in Figure 2a,b [26]. The color in the figure indicating Carbon for green, Oxygen for red, Nitrogen for blue, Hydrogen for light grey, and Hydrogen bonds for black dashed lines. While the elements of hb_1_, hb_2_, and hb_3_ indicate the hydrogen bond between molecules. Up to now, there are several solvent types which can be utilized to dissolve native chitin. This review discusses the effect of native chitin dissolution in various solvents.

#### 3.1.1. Alkali Solutions

**Figure 3:**
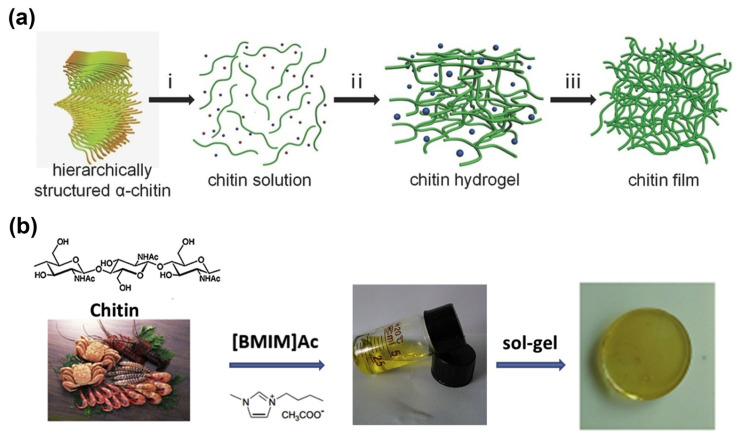
(**a**) Fabrication of chitin film: (i) prompt solubilization of chitin powder in a KOH/urea aqueous medium, (ii) a stepwise sol–gel transition followed by extensive aqueous purification produces the chitin hydrogel, (iii) drying at ambient temperature facilitates the formation of chitin film (Reproduced with permission from [28]); (**b**) a schematic representation of chitin hydrogel fabrication via dissolution in 1-butyl-3-methylimidazolium acetate ([BMIM]Ac) solvent (reproduced with permission from [32]).

One of the most used alkali solutions to dissolve chitin is alkali/urea solutions. At low temperature, chitin could be swiftly dissolved in alkali urea solution, for instance NaOH-urea solution [27] and KOH-urea solution [28]. Fang et al. reported that the intermolecular hydrogen bonds were destroyed in chitin chains and the intramolecular hydrogen bond between chitin and the aqueous solution occurred. As shown in Figure 2c, it was observed that urea played a vital role in stabilizing the chitin solution [27]. The scheme of chitin film making with KOH-urea aqueous solution was depicted in Figure 3a [28].

The freezing–thawing process is often used as a method to expedite the dissolution of chitin in alkali/urea aqueous solution [29]. Li et al. reported that a chitin hydrogel was prepared by dispersing chitin in 11 wt% NaOH-4 wt% urea aqueous solution by using the freeze–thaw process [30]. The obtained chitin hydrogel showed a high performance with the maximum efficiency of desalination up to 80% in sea water desalination application. In the experiment of Zou et al. chitin was dissolved in 20 wt% KOH-4 wt% urea aqueous solution with a freeze–thaw process [31]. The acquired chitin hydrogel has a good biocompatibility, biodegradability, anti-cell adhesion, and mechanical properties, which is becoming a promising biomaterial in the application of post-operative adhesion prevention.

Many researchers implemented chitin hydrogel preparation using alkali/urea solution due to its simplicity, cost effectiveness, and low environmental impact. However, this method requires a longer time because the freezing–thawing process is conducted in multiple cycles.

#### 3.1.2. Ionic Liquids

Ionic liquids (ILs) are salts that exist in a liquid state at room temperature. Dissolving chitins in ILs is influenced by the degree of deacetylation (DA) and molecular weight of chitin [33]. Deng and Zhang dissolved chitin into 1-butyl-3-methylimidazolium acetate ([BMIM]Ac) as illustrated in Figure 3b to prepare chitin/[BMIM]Ac gel [32]. Subsequently, the [BMIM]Ac was replaced by KOH solution to obtain chitin/KOH regenerated hydrogel, which is further used as the polymer electrolyte in supercapacitor application. The study presented that regenerated chitin hydrogel demonstrated better cyclic performance and higher capacitance compared to KOH aqueous solution. However, the process of chitin dissolution in ([BMIM]Ac) requires continuous stirring at 80 °C. Therefore, it is important to find an alternative of chitin solvent with less energy usage.

#### 3.1.3. Polar Solvents

Polar solvents are mixed with water with the aim for hydrolysis and deacetylation reaction, as well. Zhang and Yan used D-Glucosamine (GlcN) with co-solvent of γ-valerolactone (GVL), tetrahydrofuran (THF), 1,4-dioxane (DOX), dimethyl sulfoxide (DMSO), ethylene glycol dimethyl ether (EGDM), ethylene glycol diethyl ether (EGDE), and diethylene glycol diethyl ether (DGDE) [34]. The study presented that etheric solvent exhibits better performance than the other used polar solvents. Chitin was completely converted into a mixture of DGDE and water solution (4:1 *v*/*v*) with 80% GlcN in acidic condition from 100 mM sulfuric acid by heating at 175 °C for 1 h. The acid plays a role as the acid catalyst in a more efficient way when pairing with polar solvent compared to the traditional hydrolysis process in water. The study allows us to get insight into polar solvent selection strengthened by acid catalyzed conditions in chitin dissolution.

**Figure 4:**
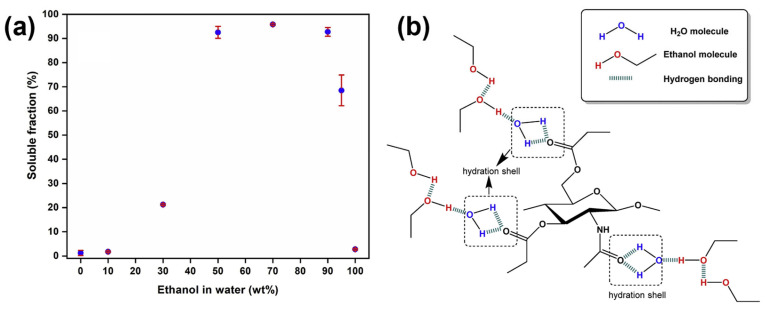
(**a**) Solubility profile of chitin propionate in ethanol/water binary solvent systems with varying ethanol concentrations; (**b**) schematic illustration of possible hydrogen bonding interactions between chitin propionate repeating units, water molecules, and ethanol molecules. Reproduced with permission from [35].

Zhong et al. prepared a chitin propionate film by dissolving chitin powder in propionic anhydride/perchloride acid mixture at 0 °C for the first 30 min then at room temperature for 2.5 h continuous stirring [35]. After water precipitation, washing, and repeated filter process, chitin propionate was obtained with superior yield of 132%. This excellent yield was obtained with the presence of a propionyloxy group into chitin propionate molecules. The chitin propionate film was subsequently fabricated via casting in an ethanol/water binary solvent system with three varying ethanol concentration conditions (90 wt%, 70 wt%, and 50 wt%). As presented in Figure 4a, the best result was obtained in binary solvent of 30 wt% water with the achievement of 96% solubility of chitin propionate. The authors successfully made chitin propionate film with more processes afterward and exhibit an excellent tensile strength to increase water-resistance in paper substrate. This work presented an improved solubility of chitin propionate in ethanol/water solvent through the bonding of each molecule as illustrated in Figure 4b, which is less toxic and provides us the opportunity to be implemented in coating, film, and ink industries.

#### 3.1.4. Deep Eutectic Solvents (DESs)

**Figure 5:**
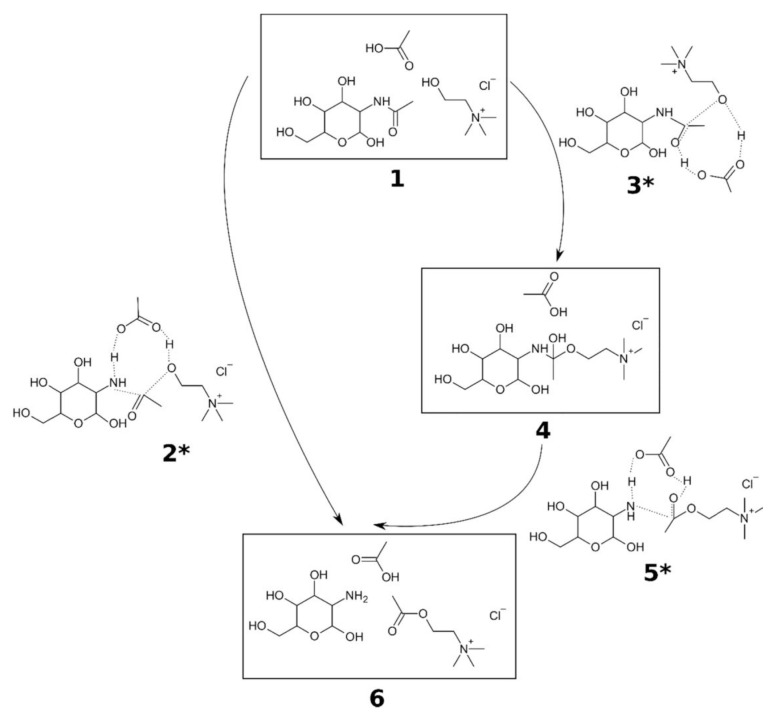
Molecular structures, including intermediates and transition states (denoted by asterisks), involved in the deacetylation of GlcNAc using [Ch]Cl:AA. The reaction may follow either a single-step pathway (1,2*,6) or a two-step pathway (1,3*,4,5*,6). A comparable reaction mechanism is observed with [Ch]Cl:OA and [Ch]Cl:MA systems. Reproduced with permission from [37].

Deep eutectic solvents (DES) is a mixture of two or more constituents that possess a lower melting point compared to its individual component [36]. Vicente et al. used four different hydrogen-bond acceptor (HBA) which are choline chloride ([Ch]Cl), choline dihydrogen citrate ([Ch]DHC), potassium bicarbonate, and potassium carbonate to six different hydrogen-bond donors (HBD) of ethylene glycol (EG), acetic acid glacial (AA), oxalic acid (OA), malic acid (MA), and citric acid (CA) monohydrate [37]. Each HBD was mixed with all HBAs, as schemed in Figure 5, by heating at 80 °C. The mechanism of using [Ch]Cl:AA (structure 1 in Figure 5) could happen in conjunctive way or two-steps. When it happened conjunctively (2*), the oxygen atom in choline attached to amidic carbon. At the same time, protonation of nitrogen occurred in acidic condition from acetic acid, facilitating C-N bond dissociation. For two-steps mechanism, the similar C-O bond occurred (3*). The protonation of amidic oxygen atom happened by the acetic acid (4). Subsequently, the C–N bond cleavage occurred through proton transfer (6). Other combination of HBD and HBA were following the similar mechanism.

Then, chitin was mixed into those solvents at several temperature variables with continuous stirring for 24 h. The experiment showed that the highest degree of deacetylation (DDA) of 40% was obtained by the combination of [Ch]Cl:MA at 120 °C for 24 h. The study demonstrates that DES effectively enhances the reactivity of chitin, facilitating its dissolution and subsequent processing. Although the achievement is less than 50%, there are opportunities to make more combinations of DES to increase the deacetylation of chitin.

The comparison of those four methods in preparation of chitin-based hydrogels is summarized in Table 1 below, including their advantages and disadvantages.

The selection of the solvent system should be based by criteria such as dissolution efficiency to ensure effective cleavage of hydrogen bond in chitin. processing condition to determine the energy consumption and scalable production, cost and availability for better accessibility in industrial application, compatibility with additives to tailor the hydrogel properties, and solvent recyclability for green processing.

Overall, each solvent system presents distinct advantages and limitations. Alkali-based systems are cost-effective and environmentally friendly but often require time-consuming freeze–thaw cycles. Ionic liquids enable efficient dissolution and improved material performance, although they involve higher cost and energy consumption. Polar solvents and deep eutectic solvents offer alternative pathways with tunable reactivity and greener profiles, yet their scalability and efficiency remain under development. Therefore, the selection of solvent system should be guided by the targeted application, considering factors such as processing efficiency, environmental impact, and desired hydrogel properties.

### 3.2. Preparation of Hydrogels from Chitin Derivatives

#### 3.2.1. Preparation of Hydrogels from Chitosan and Its Derivatives

When chitin structure is modified by removing an acetyl group in its C-2 position of acetamide and forming an amino group, chitosan is formed. Chitosan is the result of the chitin deacetylation process. Other functional groups can be introduced to the chitosan group and will provide different chemical and physical properties. Demirtas et al. prepared alginate, alginate-hydroxyapatite (alginate-HA), chitosan and chitosan-hydroxyapatite (chitosan-HA) hydrogels by thermal crosslinking reaction at 37 °C to investigate bio-ink characterization [38]. The result showed the chitosan and chitosan-HA hydrogels exhibited increased viscosity and higher elastic modulus compared to alginate-based hydrogels. In addition, the introduction of hydroxyapatite (HA) functional group provided a superior performance compared to the non-HA hydrogels for both alginate-based and chitosan-based hydrogels. This study proved that chitosan and chitosan-HA hydrogels were successfully created as bioprinting solutions for bone tissue engineering application.

#### 3.2.2. Hydrogels from Other Chitin Derivatives

Liu et al. introduced carboxymethyl group to chitin to form carboxymethyl chitin (CMCH) hydrogel [39]. CMCH was prepared by the etherification process of hydroxyl group in chitin with sodium monochloroacetate in NaOH/urea solution. Three variables of CMCHs were used with the ratio of chitin/sodium monochloroacetate of 0.14, 0.21, and 0.31, respectively. The result pointed out that all three CMCHs are soluble in 0.1 M NaOH solution, in which the pristine chitin cannot dissolve in it. It can be concluded that introducing carboxyl groups could improve the dissolution of modified chitin.

**Figure 6:**
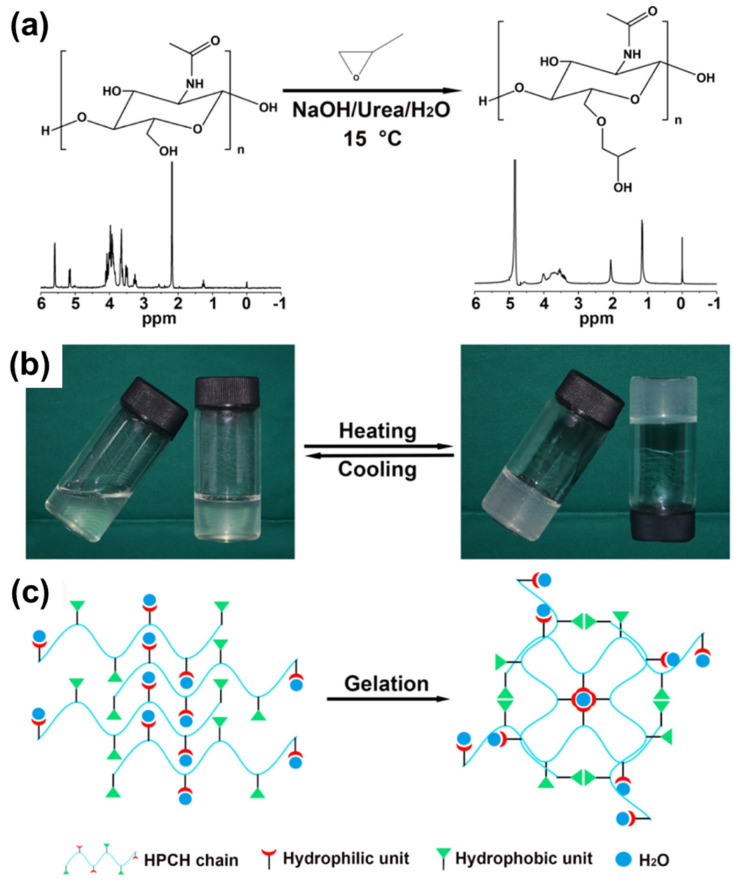
Synthesis and thermoresponsive behavior of HPCH hydrogel: HPCH is prepared in an aqueous NaOH/urea solution. (**a**) ^1^H NMR spectrum of HPCH was obtained following hydrolysis in 10% (*w*/*v*) DCl/D_2_O at 25 °C, alongside a reference ^1^H NMR spectrum of chitin hydrolyzed in 37% DCl at 50 °C; (**b**) Photographic evidence demonstrates the thermoreversible sol–gel transition of a 3 wt% HPCH solution in PBS between 4 °C and 37 °C; (**c**) A schematic diagram illustrates the physical crosslinking mechanism. Reproduced with permission from [40].

Another study by Xu et al. was done by incorporating hydroxypropyl group to chitin to form hydroxypropyl chitin (HPCH) hydrogel, as shown in Figure 6a [40]. HPCH was prepared in NaOH/urea aqueous solution. Then, it was further processed to obtain a moldable thermosensitive HPCH. The details of sol–gel transition and gelation scheme were depicted in Figure 6b,c. The result demonstrated that HPCH was successfully created, with excellent biocompatibility and low cytotoxicity for in vitro and in vivo 3D cartilage regeneration for clinical treatment application.

**Figure 7:**
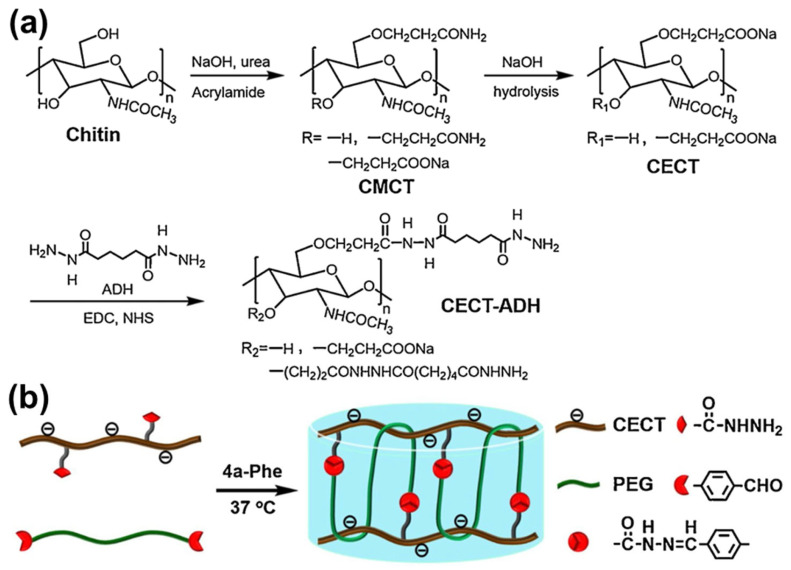
Fabrication of CECT-ADH and CECT-ADH/PEG-DA hydrogels. (**a**) Synthesis pathway of CECT-ADH; (**b**) Schematic representation of CECT-ADH/PEG-DA hydrogel formation through dynamic acylhydrazone crosslinking interactions. Reproduced with permission from [15].

On the other hand, Yang et al. introduced a carboxyethyl group to chitin to form carboxyethyl chitin (CECT) [15]. It was further reacted with adipic dihydrazide (ADH) in MES aqueous solution. Dialysis and freeze-drying were done afterwards to obtain adipic dihydrazide-grafted carboxyethyl chitin (CECT-ADH) through synthetic route depicted in Figure 7a. The hydrogel, which is illustrated in Figure 7b, exhibited a good self-healing and self-adaptation, in vitro/in vivo enzymatic degradability which is potential as delivery agent for drug release in vivo. These modifications highlight the importance of chemical functionalization in improving solubility, tunability, and application-specific performance of chitin-based hydrogels.

### 3.3. Preparation of Hydrogels from Nano Chitin

**Figure 8:**
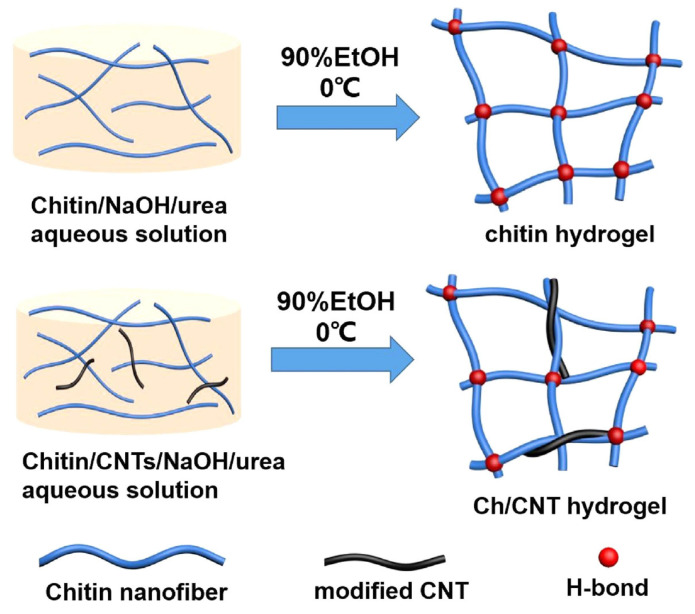
Illustrative schematic of the fabrication process for chitin and chitin/carbon nanotube (Ch/CNT) composite hydrogels. Reproduced with permission from [41].

The study of introducing nanoparticles to chitin hydrogel was also something intriguing to be developed. Wu et al. prepared chitin carbon nanotube (Ch/CNT), as shown in Figure 8, by dissolving native chitin in a mixture of sulfuric acid 70% and nitric acid 65% (3:1 *v*/*v*) by heating at 60 °C for 2 h [41]. Then, the dialysis process was repeatedly done until the pH is neutral and continued by freezing-drying process. The functionalized CNTs were dispersed in an aqueous NaOH/urea solution and combined with native chitin at three distinct concentrations (1 wt%, 3 wt%, and 5 wt%). The resulting mixtures, labeled as Ch/CNT1, Ch/CNT2, and Ch/CNT3, respectively, were processed through a freeze–thaw method to form composite hydrogels. The result showed that Ch/CNT manifested a good strengthening and toughening effect compared to chitin hydrogel. This is mainly due to higher crosslinking density in Ch/CNT hydrogel. All Ch/CNT hydrogels displayed a good tensile strength and elongation which are a good prospect for peripheral nerve regeneration. The incorporation of nanomaterials further enhances mechanical strength and functionality, indicating strong potential for advanced biomedical applications.

## 4. Physicochemical Characteristics of Chitin-Based Hydrogels

### 4.1. Mechanical Properties

**Figure 9:**
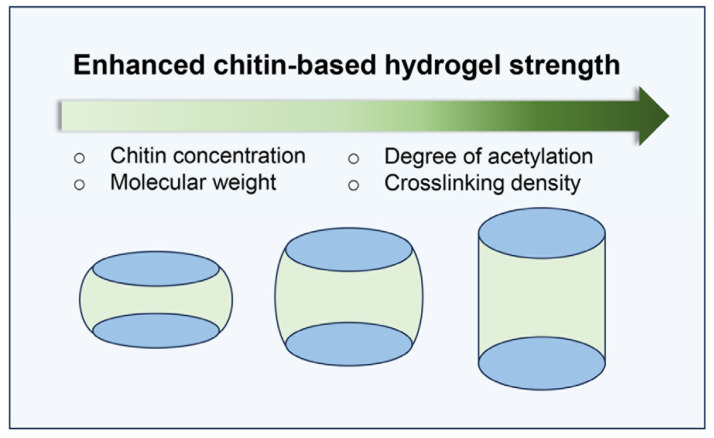
Schematic of factors enhancing chitin-based hydrogel strength.

Naturally, chitin exists as part of a composite structure with proteins, minerals, and pigments. The choice of extraction method, whether mechanical, chemical, or enzymatic, not only removes these associated components but also affects the molecular structure of chitin, which in turn influences its mechanical properties [42]. In this context, key properties such as tensile strength, compressive modulus, shear rheology, elasticity, and structural integrity are influenced by factors including polymer concentration, molecular weight, degree of acetylation (DA), crosslinking method, and processing conditions (Figure 9).

Chitin’s β-(1→4)-linked N-acetylglucosamine structure contributes to a semi-crystalline backbone, which imparts inherent rigidity and strength. Increasing the polymer concentration and using high molecular weight chitin typically enhance strength of hydrogel by promoting greater chain entanglement and intermolecular interactions [43,44,45]. While a lower degree of acetylation reduces hydrogen bonding, chain stability and crystallinity, resulting in a more flexible and tougher hydrogel network [46,47,48].

The mentioned inherent flexibility, however, must be supported by an appropriate crosslinking strategy to achieve the desired mechanical properties. Physically crosslinked chitin-based hydrogels are typically formed through hydrogen bonding or ionic interactions, often involving multivalent ions. These physical gels are generally soft, elastic, and exhibit reversible behavior, which can be advantageous for applications requiring conformability and responsiveness [49,50]. However, their reliance on weak intermolecular forces often results in poor mechanical strength and limited stability under physiological conditions. In contrast, recent studies report that chemically crosslinked chitin-based hydrogels synthesized using crosslinking agents such as 1,4-butanediol diglycidyl ether (BDDE) [31], genepin [51], and glutaraldehyde [52], form stable covalent networks that significantly enhance mechanical strength and durability. Consequently, it is more suitable for load-bearing biomedical applications, including drug release, cartilage regeneration and tissue scaffolds, where enhanced strength and durability are required [31,49,50,51,52]. In addition, processing condition parameters such as polarity [53], gelation temperature [53,54], and pH condition [55,56], may influence the internal microstructure and crosslinking density of the chitin-based hydrogel, which affect its mechanical performance. Moreover, the incorporation of nanoscale reinforcements like carbon nanotube (CNT) [57] and poly(3,4-ethylenedioxythiophene) nanoparticles (PEDOT NPs) [58] enhances stress distribution within the polymer network, leading to improved mechanical strength and elastic flexibility.

To quantitatively evaluate these mechanical attributes and understand how structural factors, crosslinking strategies, and processing conditions translate into performance, rheological characterization is employed as a key analytical tool [32,59,60,61,62]. Chitin-based hydrogels demonstrate diverse rheological properties depending on the solvent system and formulation strategy [32,59,60,61,62]. Hydrogels prepared using calcium chloride–methanol exhibit elastic behavior with a storage modulus (G′) of 9.2 kPa, where G′ exceeds the loss modulus (G″), indicating solid-like characteristics. These gels are pseudoplastic, showing shear-thinning behavior with a phase angle below ten degrees, confirming high elasticity and good injectability [59]. In ionic liquid systems, the G′ value increases significantly up to 112 kPa, retaining shear-thinning and self-recovery properties after deformation [32]. NaOH/urea-based gels have a moderate G′ around 21.8 kPa, also displaying G′ > G″ and shear-thinning behavior, though gelation is delayed in the presence of water [60]. Deep eutectic solvent (DES)-derived hydrogels likewise demonstrate shear-thinning rheology, with mechanical strength tunable via processing conditions [61]. LiCl/DMAc-based hydrogels show pseudoplastic or Newtonian behavior, depending on polymer concentration, and exhibit temperature-sensitive viscosity [62]. Across systems, higher G′ values and G′ > G″ consistently correlate with better mechanical strength and stability, making these hydrogels suitable for many applications requiring elasticity, injectability, and structural integrity [32,59,60,61,62].

### 4.2. Microstructural Morphology

The pore structure and surface features of chitin-based hydrogels critically influence their mechanical properties, swelling behavior, and suitability for some applications. These characteristics are primarily dictated by the crosslinking mechanism, processing conditions, and drying techniques [45,63,64,65,66,67,68,69]. For instance, physical cross-linked chitin-based hydrogels coagulated in alkaline urea solutions exhibit enhanced structural uniformity, characterized by smoother surface topography, reduced pore diameter, and increased network density, compared to those precipitated in neutral or weakly polar solvents such as water or ethanol [63,64]. These microstructural refinements not only contribute to improved mechanical properties but also enhance optical transparency by minimizing light scattering through irregular interfaces and large pores [64]. In chemically crosslinked systems, the pore size generally falls within a few micrometers upon freeze-drying, with finer structures achievable through increased polymer concentration and crosslinking density [45,65].

The drying technique significantly influences the microstructure of materials [66]. Lyophilization (freeze-drying) typically promotes the formation of highly interconnected macroporous networks within chitin-based hydrogel through ice sublimation, which helps preserve the original three-dimensional structure [67]. In comparison, supercritical drying, which involves solvent exchange followed by depressurization beyond the critical point, enables the production of nanoporous with high surface area and minimal structural collapse [68]. Moreover, hydrogels derived from chitin nanowhiskers (ChNWs) exhibit uniformity and optical clarity, primarily due to physical entanglement and strong hydrogen bonding interactions within the polymer network [69]. The gelation process can be triggered by external stimuli including ultrasonication, pH modulation, or solvent displacement, leading to the development of robust three-dimensional networks even at relatively low concentrations. By systematically adjusting processing parameters, the pore structure of chitin-based hydrogels can be finely controlled to enhance their functionality for specific end-use applications.

### 4.3. Swelling Capacity and Water Retention

Chitin-based hydrogels exhibit high swelling capacity and water retention due to the presence of hydrophilic functional groups such as hydroxyl and acetamido moieties, which facilitate hydrogen bonding with water [47]. The swelling behavior is primarily influenced by crosslinking density, pore architecture, and the method of drying [31,54,56,62]. Low crosslinking densities and open, interconnected pore networks enhance water absorption, while highly crosslinked systems restrict swelling [54,70]. Freeze-dried and supercritically dried chitin hydrogels preserve porous structures, allowing rehydration and swelling ratios exceeding 60 g/g [71] and 106–107 g/g [56]. In contrast, air- or oven-dried samples tend to collapse structurally, reducing water retention.

Molecular weight significantly influences performance, wherein chitin with higher molecular weight demonstrates increased water uptake as a result of enhanced polymer chain entanglement and elevated hydrophilicity [72]. Additionally, environmental conditions such as pH and ionic strength affect swelling, where alkaline or neutral pH favors higher absorption, particularly in chitosan-rich systems derived from partially deacetylated chitin [56]. Tailoring swelling capacity or water uptake in chitin-based hydrogels enables their use in diverse applications. High swelling is ideal for wound dressings and absorbents, while controlled hydration suits drug delivery systems. By adjusting structure and processing, chitin hydrogels can be engineered for specific moisture-handling needs.

### 4.4. Degradation Behavior

Due to their polysaccharide backbone composed of β-(1→4)-linked N-acetylglucosamine units, chitin-based hydrogels exhibit inherent biodegradability. These β-(1→4) glycosidic linkages serve as the primary cleavage sites for enzymatic hydrolysis by naturally occurring enzymes such as chitinase, and to a lesser extent lysozyme, which break the polymer chains into smaller oligosaccharides and monomers. The degradation process is influenced by environmental conditions, including pH, temperature, and enzyme concentration, which affect enzymatic activity and hydrolysis efficiency. Under physiological conditions, this allows the hydrogel matrix to degrade gradually into non-toxic byproducts, making chitin hydrogels particularly suitable for biomedical applications where material resorption is desired, such as wound healing [73,74,75,76,77] or drug delivery [78,79].

The biodegradation rate of chitin hydrogels can be controlled by tuning key structural parameters. A lower degree of acetylation (DA) leads to increased enzymatic accessibility and faster degradation, as it reduces the crystalline regions that typically hinder enzyme penetration [48,80]. Similarly, hydrogels with lower crosslinking density and higher porosity allow easier water and enzyme diffusion into the network, accelerating breakdown. Physically crosslinked chitin hydrogels tend to degrade more quickly than chemically crosslinked ones due to the absence of strong covalent bonds within the network.

Chemical modifications of chitin, including processes like carboxymethylation or hydroxypropylation, can further enhance biodegradability by increasing hydrophilicity and disrupting the crystalline structure [70,81,82]. These modifications not only improve solubility and gelation but also enable the design of hydrogels with adjustable degradation rates, depending on the application. For example, rapidly degrading hydrogels may be useful for short-term drug release [83,84,85], while slower-degrading systems are better suited for tissue scaffolding [86]. Overall, the biodegradability of chitin-based hydrogels is a highly tunable and application-relevant property, offering a sustainable and safe alternative for transient biomedical and environmental uses.

### 4.5. Stimuli-Responsive Behavior

Stimuli-responsive behavior is an important physicochemical characteristic of chitin-based hydrogels, enabling them to respond dynamically to external stimuli such as pH, temperature, ionic strength, and electric or magnetic fields. These responses are typically manifested through changes in swelling behavior, mechanical properties, or network structure [78,87].

The responsiveness of chitin-based hydrogels is closely related to their chemical composition, degree of acetylation, and crosslinking structure. For instance, pH-responsive behavior arises from the ionization of functional groups, leading to variations in hydrogel swelling and stability under different pH conditions, while thermoresponsive systems exhibit sol–gel transitions depending on temperature [78,87]. In addition, the incorporation of nanoparticles or conductive components can further introduce responsiveness to external stimuli such as electric or magnetic fields [88,89]. By tailoring these structural and compositional factors, chitin-based hydrogels can be engineered to exhibit controlled and tunable responses, enabling tunable behavior that can be further utilized in various advanced applications.

## 5. Advanced Applications of Chitin-Based Hydrogels

Owing to their intrinsic biocompatibility, enzymatic degradability, high moisture retention, and structural adaptability, hydrogels derived from chitin have emerged as versatile materials across multiple disciplines, as shown in Table 2.

### 5.1. Superabsorbent

**Figure 10:**
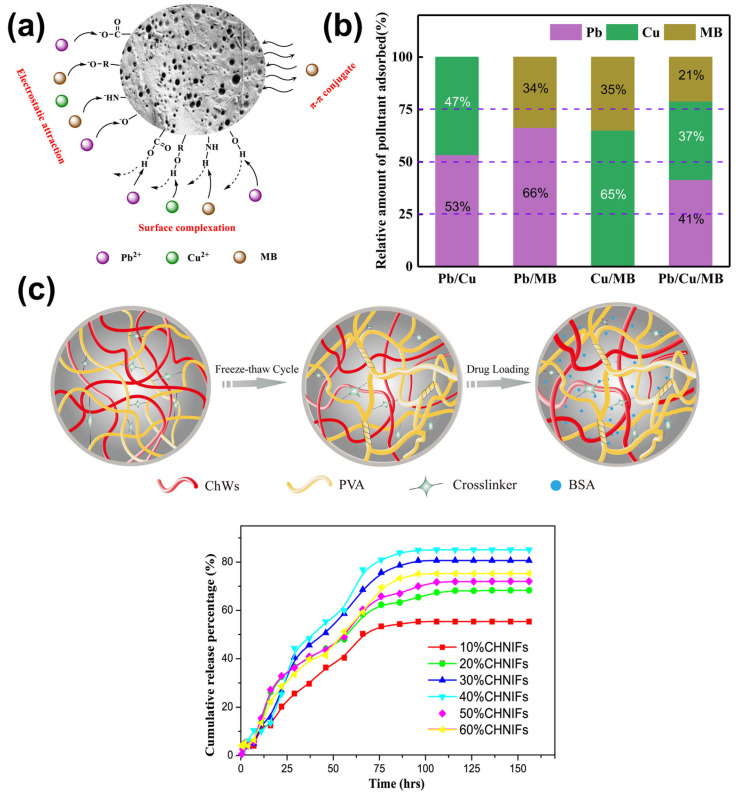
(**a**) Schematic representation of the adsorption pathways for Pb^2+^, Cu^2+^, and MB; (**b**) Selective adsorption behavior of the adsorbent in multi-component pollutant system. Reproduced with permission from [92]; (**c**) Diagram showing chitin-based hydrogel composites and the corresponding BSA release profile at 25 °C. Reproduced with permission from [95].

The absorption capacity of chitin-based hydrogels is one of their most important characteristics, driven by the presence of hydrophilic groups such as hydroxyl (-OH) and acetamido (-NHCOCH_3_) moieties in the chitin backbone. These groups facilitate strong hydrogen bonding with water molecules, resulting in high swelling ratios and water retention. In aqueous environments, chitin hydrogels act as superabsorbent materials [89], making them suitable for wound dressings, where they maintain a moist healing environment and absorb wound exudates [86,91]. This performance can be further enhanced through composite design, where the incorporation of inorganic components such as kaolin improves structural stability and promotes rapid hemostasis via synergistic fluid absorption and coagulation activation, thereby strengthening their potential for advanced wound care applications [113]. Their three-dimensional (3D) porous structure provides a large surface area for fluid uptake, while also allowing gas exchange and nutrient transport. In environmental applications, derivate chitin-based hydrogels serve as biosorbents capable of removing pollutants such as heavy metals (e.g., Cu^2+^, Pb^2+^) and dyes from wastewater [92,93,94]. For example, Figure 10a outlines the conceptual mechanism by which the chitin-based hydrogel interacts with contaminants [92]. The adsorption process is governed by the synergistic action of hydroxyl, carboxyl, and amino functional groups embedded within the hydrogel matrix. These groups facilitate strong interactions with metal ions (Pb^2+^, Cu^2+^) and dyes (MB) through mechanisms such as ion exchange, electrostatic attraction, and chelation. Notably, chemical modification of chitin or its derivatives through carboxymethylation significantly enhances interaction efficiency, thereby greatly improving the hydrogel’s capacity for contaminant adsorption and retention. As illustrated in Figure 10b, under competitive adsorption conditions with multiple coexisting pollutants, the hydrogel exhibits a clear preference for capturing Pb^2+^ over Cu^2+^ and MB. This selective behavior is largely influenced by the higher electronegativity and stronger binding affinity of Pb^2+^, which facilitates its more effective interaction with the functionalized hydrogel surface [92]. In this regard, chitin-based hydrogels show excellent reusability and regeneration potential, making them sustainable and cost-effective for repeated cycles of adsorption–desorption in water treatment systems. Overall, the absorption and adsorption capabilities of chitin-based hydrogels make them highly versatile for both biomedical and environmental applications.

### 5.2. Controlled Delivery Systems

With their biodegradable nature, non-cytotoxicity, and adjustable physical properties, chitin-based hydrogels are well-suited for use in controlled drug delivery systems [96,97]. The porous matrix of chitin-based hydrogels allows for effective encapsulation of both hydrophilic and hydrophobic drug molecules due to their tunable physicochemical properties, such as degree of deacetylation and crosslinking density. The drug release kinetics are primarily governed by diffusion through the hydrated gel layer, polymer degradation via enzymatic action (e.g., lysozyme in biological environments), and swelling-controlled mechanisms, which are influenced by pH-dependent solubility and gel-layer dynamics [96,98]. For instance, chitosan (derived from chitin) forms a mucoadhesive gel layer in acidic conditions, enabling sustained release, while its erosion and degradation further modulate release rates in physiological environments [99,100].

Stimuli-responsive chitin derivatives, such as carboxymethyl chitin (CMCH) and hydroxypropyl chitin (HPCH), offer advanced control over drug [78,87]. Zheng et al. demonstrated that CMCH forms pH-sensitive hydrogels where ionization of carboxyl groups induces swelling at neutral or alkaline pH, suitable for targeted delivery in intestinal environments [78]. On the other hand, HPCH hydrogels exhibit thermoreversible gelation, forming a gel at physiological temperature, ideal for injectable in situ gel systems. This property facilitates site-specific drug delivery with minimal procedural invasiveness [78]. While these systems provide effective stimuli-responsive delivery, they are often limited to passive release and single-stimulus responsiveness. In contrast, thermosensitive chitin-based hydrogels integrating photothermal and antibacterial functions enable controlled release under mild conditions (~48 °C), while enhancing antibiofilm activity and wound healing, highlighting their potential for treating multidrug-resistant infections [114]. Furthermore, by blending chitin with other polymers [54,95] or incorporating nanoparticles [88,89], multifunctional hydrogel systems can be created with enhanced mechanical stability, drug loading capacity, and responsive behavior to pH, temperature, or ionic strength. For example, chitin-based hydrogel composites formed by blending chitin nanowhiskers (ChWs) with poly(vinyl alcohol) (PVA) exhibit an interconnected porous network capable of efficiently encapsulating and gradually releasing biomolecules such as BSA, as illustrated in Figure 10c [95]. The corresponding release profile reveals a two-phase mechanism: an initial burst release driven by rapid swelling and diffusion, followed by a more gradual and sustained release as equilibrium is approached. Among the tested formulations, the hydrogel containing 40% ChWs demonstrated the most effective release performance, likely due to its well-balanced combination of porosity and mechanical strength, which highlights their strong potential for use in implantable drug delivery platforms. These systems demonstrate the growing potential of chitin-based hydrogels as multifunctional platforms for controlled and responsive therapeutic delivery.

### 5.3. Stimuli-Sensitive Systems

Chitin-based hydrogels can be engineered to exhibit stimuli-sensitive behavior, enabling them to undergo physical or chemical changes in response to external stimuli such as pH, temperature, magnetic field or electric field, as shown in Table 3 [15,87,101,102,103,104].

Pure chitin lacks inherent stimuli-responsiveness; however, chemical modifications or blending with responsive polymers can impart these functionalities. For instance, the pH-responsiveness is commonly introduced through carboxymethylation of chitin, producing carboxymethyl chitin (CMCH). The carboxyl groups in CMCH ionize in neutral or alkaline media, leading to electrostatic repulsion within the network and enhanced swelling [78,87]. This enables pH-triggered drug release, ideal for targeting specific regions of the gastrointestinal tract. Building upon this concept of environmental responsiveness, temperature sensitivity can be imparted by grafting chitin or its derivatives with thermoresponsive polymers such as poly(N-isopropylacrylamide) (PNIPAm) [101] and poly (di(ethylene glycol) methyl ether methacrylate) (PMEO_2_MA) [102]. These hydrogels swell below their lower critical solution temperature (LCST) and shrink above it, allowing on–off switching of drug release at body temperature. Further expanding their functionality, magnetic responsiveness can be achieved by embedding magnetic nanoparticles (e.g., Fe_3_O_4_) into the hydrogel structure [87]. These composite hydrogels respond to external magnetic fields, enabling remote actuation and spatially controlled release, which are especially beneficial for non-invasive targeting in biomedical systems.

In parallel, electric field-sensitive chitin-based hydrogels have also been developed to respond to electrical stimuli, offering additional potential for on-demand therapeutic control. For instance, chitin derived hydrogel from *Hericium erinaceus* demonstrates reversible bending and swelling under electric fields, driven by ion migration and osmotic gradients [103]. This behavior enabled controlled curcumin release and showcased promising electromechanical responsiveness for biomedical applications such as soft actuators and electrically triggered drug delivery. In another study, acrylamide-modified chitin (AMC) exhibited dual responsiveness to both pH and electric signals. This water-soluble chitin derivative could undergo sol–gel transitions in response to electrochemically induced pH changes or redox-active ions (e.g., Fe^3+^/Fe^2+^), enabling programmable protein entrapment and release directly on electrode surfaces [104].

### 5.4. Energy Device and Smart Sensors

**Figure 11:**
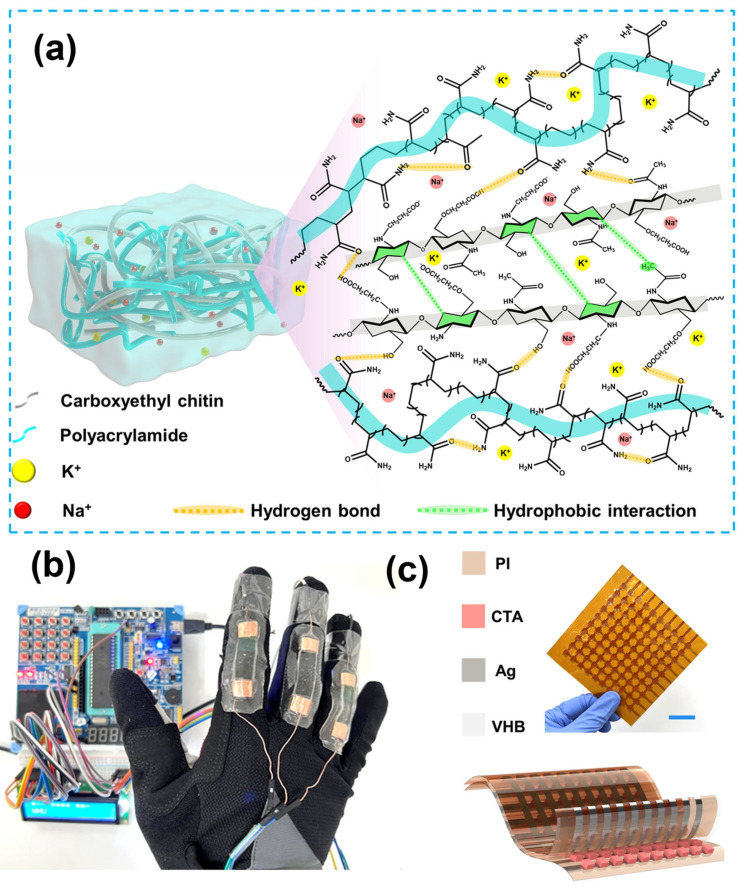
(**a**) Schematic of carboxyethyl chitin-polyacrylamide interaction in composites hydrogels; (**b**) Photograph of a human–machine interface device utilizing carboxyethyl chitin/polyacrylamide hydrogels; (**c**) Integrated carboxyethyl chitin/polyacrylamide hydrogel-based electronic skin array. Reproduced with permission from [105].

Beyond biomedical use, chitin-based hydrogels and their derivatives have gained increasing attention in the development of energy devices and smart sensor platforms. Their intrinsic biocompatibility, biodegradability, and mechanical flexibility make them ideal for wearable electronics, energy harvesting systems, and environmental sensing applications. For instance, chitin hydrogel fabricated via non-freezing dissolution in KOH/urea systems have been employed as tribopositive layers in triboelectric nanogenerators (TENGs), achieving high output voltages (up to 182.4 V) and power densities over 1.25 W/m^2^, thus enabling self-powered sensing systems for tactile recognition and physiological monitoring [21]. In addition, carboxyethyl chitin/polyacrylamide hydrogels have been developed with ultra-stretchability (>1500%), high transparency, and strong adhesion, making them well-suited as electronic skins capable of detecting fine strains and pressures as described in Figure 11a, which illustrates their molecular interaction network and structural design [105]. These hydrogels demonstrate high gauge factors (~18.5) and broad sensing ranges, supporting applications in human–machine interfaces (Figure 11b), including Morse code recognition via finger gestures, and spatially resolved pressure sensing via integrated electronic skin (e-skin) arrays for wearable electronics (Figure 11c) [108].

Further advancement includes the integration of chitin nanoparticle into deep eutectic eutectogels, resulting in self-healing, temperature-tolerant, and ultra-stretchable sensors that can conform to dynamic surfaces like human joints or internal organs [106,107]. These multisignal wearable sensors exhibit high mechanical durability and maintain sensing function under extreme environments, expanding their use for long-term health monitoring. Moreover, amide-modified chitin-based hydrogels loaded with ε-polylysine and Al^3+^ ions provide antibacterial, adhesive, and electrically conductive properties [108]. These hydrogels function effectively as real-time motion sensors, capable of detecting both subtle and gross human movements while preserving long-term performance through self-healing mechanisms. Taken together, these innovations highlight the vast potential of chitin-derived materials in building eco-friendly, biocompatible, and high-performance components for self-powered wearable electronics and soft intelligent devices.

### 5.5. Tissue Engineering

Chitin-based hydrogels have attracted considerable attention in tissue engineering due to their intrinsic biocompatibility, biodegradability, and structural similarity to the extracellular matrix (ECM). Rather than functioning solely as passive scaffolds, recent developments emphasize their role as tunable platforms capable of directing cellular behavior through controlled architecture and composition. For instance, the introduction of anisotropic structures, as demonstrated in chitin–tannic acid/brushite hydrogels, enables directional cell migration and enhances osteogenic differentiation, highlighting the importance of structural alignment in guiding tissue regeneration [109]. Similarly, the integration of chitin whisker/chitosan liquid crystal phases into 3D-printed scaffolds allows the formation of bone-like ECM microenvironments with hierarchical organization, which not only supports osteogenesis but also promotes vascularization [110]. These studies collectively suggest that mimicking the structural complexity of native tissues, including hierarchical organization and anisotropic features, is a key factor in improving regenerative outcomes.

In parallel, reinforcing strategies such as the incorporation of chitin whiskers have been shown to address one of the major limitations of hydrogel systems, namely their insufficient mechanical stability. Enhanced mechanical strength and anti-deformation properties are critical for maintaining scaffold integrity during long-term cell culture and tissue formation [111]. However, these improvements often involve a trade-off between mechanical robustness and matrix flexibility, which may influence cell behavior and diffusion properties. Extending beyond bone-related applications, hybrid chitin-based hydrogels combining bioactive components such as fibrin or Matrigel further demonstrate the potential to replicate both the biochemical and mechanical aspects of native ECM. These systems have been shown to support endothelial cell spreading, promote angiogenesis, and enable the fabrication of functional cardiac tissue constructs via 3D bioprinting [112]. Despite these advances, challenges remain in achieving precise control over multi-scale architecture, long-term stability, and reproducibility. The current body of work indicates that the future of chitin-based hydrogels in tissue engineering lies in balancing structural design, mechanical performance, and biological functionality to create more predictive and application-specific scaffolds.

## 6. Conclusions and Perspectives

The number of studies on chitin hydrogels has expanded significantly due to its remarkable advantages of abundant biopolymer, biocompatibility, degradability, non-toxicity, and ability to be chemically modified. The limitation of poor solubility in native chitin has driven extensive research into various hydrogel preparation approaches, including solvent-based systems, structural modification to generate derivatives, and incorporation of nanomaterials. While these strategies have improved processability and functionality, each method presents its own benefits and drawbacks. However, despite these advances, chitin-based hydrogels still face several challenges, including limited mechanical strength, processing complexity, and the need for chemical modification to achieve desired properties, which may limit their practical applications.

Future development should therefore focus on reducing energy consumption in hydrogel preparation to support industrial application. This is particularly critical as many current methods, such as freeze–thaw cycles, high-temperature dissolution, and ionic liquid-based systems, require significant energy input and involve complex processing steps. Addressing these challenges will not only improve process efficiency but also enhance the overall performance and practicality of chitin-based hydrogels.

With these improvements, chitin hydrogels are expected to further demonstrate their remarkable mechanical properties and high swelling capacity and retention, making them versatile in various applications in drug delivery systems, stimuli responsive capability, biosensors and electrodes. We believe that this review presents a compact understanding that might provide insight for future chitin-based hydrogels processing to improve its chemical and physical properties.

## Figures and Tables

**Table 1:** Various preparations and methods of chitin-based hydrogels.

Hydrogel Preparation	Methods	Advantages	Disadvantages	References
Alkali solutions	Chitin dissolvation in alkali/urea solutions at low temperature	Simple, cost-effective, and low environmental impact	Required longer time from multiple cycles	[27,28,29,30,31]
Ionic liquids	Chitin dissolvation in ionic liquids at temperature of 80 °C with continuous stirring	Enhances solubility in a mild processing condition	Higher cost and energy usage	[32,33]
Polar solvents	Chitin dissolvation in polar solvents at temperature of 175 °C in acidic condition	Improved interaction with functional group, better compatibility with co-solvent systems, improved material properties	Different solvents determine the performance depending on the polarity	[34,35]
DESs	Chitin dissolvation in a mixture of two of more constituents at temperature of 80 °C resulting in a lower melting point that the individual components	Enhances the reactivity of chitin	The resulting degree of deacetylation is low	[36,37]

**Table 2:** Multifunctional applications of chitin-based hydrogels.

Application Area	Mechanism/Functionality	Materials/Modifications	Key Features	Example Use/References
Superabsorbents	Absorb large amounts of water or fluids via hydrogen bonding of hydrophilic groups	Native chitin/modified derivatives (e.g., carboxymethyl chitin)	High swelling ratio, porosity, moisture retention	Wound dressing, wastewater treatment [86,90,91,92,93,94]
Controlled delivery systems	Encapsulation and timed release of drugs via swelling, diffusion, degradation	CMCH, HPCH, chitosan, thermosensitive gels	pH- or temperature-responsive release, biocompatibility, biodegradability	Intestinal or injectable drug release [54,78,87,88,89,95,96,97,98,99,100]
Stimuli-sensitive systems	Respond to pH, temperature, magnetic/electric fields to modulate hydrogel properties	CMCH, HPCH, AMC, Fe_3_O_4_-chitin composites	On–off release, reversible swelling, electro-/magneto-responsive	pH-triggered delivery, electric-actuated release [15,87,101,102,103,104]
Energy devices	Generate electricity from mechanical motion via triboelectric effect	Chitin hydrogels (e.g., KOH/urea-prepared)	High voltage output, flexibility, durability	Triboelectric nanogenerator (TENG) [21,105]
Smart sensors	Detect strain, pressure, or motion by changes in electrical or physical properties	Carboxyethyl chitin/PAM, magnetic/electroactive composites	Stretchability, transparency, conductivity, self-healing	Electronic skin, motion sensors [106,107,108]
Tissue engineering	Cell adhesion, proliferation, differentiation; ECM mimicry; support of tissue regeneration	Native or chemically modified chitin hydrogels (e.g., chitin, chitosan derivatives, composites with nanowhiskers or fibrin)	Biocompatible, biodegradable porous 3D network; tunable mechanics and degradation	3D cell culture scaffolds; bone and vascular regeneration [109,110,111,112]

**Table 3:** Stimuli-sensitive chitin-based hydrogels.

Stimulus	Modification/Composite	Response Mechanism	References
pH	Carboxymethyl chitin (CMCH)	Ionization of carboxyl groups in increases swelling in neutral/alkaline pH	[15,87]
Temperature	Poly(diethylene glycol methyl ether methacrylate) (PMEO_2_MA)-or PNIPAm-grafted chitin	Thermoreversible gelation at physiological temperature (in situ gel formation)	[101,102]
Magnetic Field	Fe_3_O_4_ nanoparticles embedded in chitin hydrogel	Magnetic actuation and spatially controlled release	[87]
Electric Field	Hericium erinaceus-derived chitin hydrogel	Ion migration causes reversible bending/swelling	[103]
Dual (pH/Temp)	Acrylamide-modified chitin (AMC)	Sol–gel transitions triggered by electrochemical pH changes or redox ions (e.g., Fe^3+^/Fe^2+^)	[104]

## Data Availability

The original contributions presented in the study are included in the article and further inquiries can be addressed to the corresponding author.

## Abbreviations

The following abbreviations are used in this manuscript:

ADH	Adipic dihydrazide
AMC	Acrylamide-modified chitin
BDDE	1,4-Butanediol diglycidyl ether
BSA	Bovine serum albumin
CECT-ADH	Adipic dihydrazide-grafted carboxyethyl chitin
ChNWs	Chitin nanowhiskers
CMCH	Carboxymethyl chitin
CNT	Carbon nanotube
DA	Degree of acetylation
Fe_3_O_4_	Iron oxide (magnetite)
G’	Storage modulus
G″	Loss modulus
HA	Hydroxyapatite
HPCH	Hydroxypropyl chitin
LCST	Lower critical solution temperature
MB	Methylene blue
PAM	Polyacrylamide
PEDOT NPs	Poly(3,4-ethylenedioxythiophene) nanoparticles
PMEO_2_MA	Poly(di(ethylene glycol) methyl ether methacrylate)
PNIPAm	Poly(N-isopropylacrylamide)
PVA	Poly(vinyl alcohol)
TENG	Triboelectric nanogenerator
